# Phytohormonal crosstalk modulates the expression of miR166/165s, target *Class III HD-ZIPs*, and *KANADI* genes during root growth in *Arabidopsis thaliana*

**DOI:** 10.1038/s41598-017-03632-w

**Published:** 2017-06-13

**Authors:** Archita Singh, Shradha Roy, Sharmila Singh, Shabari Sarkar Das, Vibhav Gautam, Sandeep Yadav, Ashutosh Kumar, Alka Singh, Sukanya Samantha, Ananda K. Sarkar

**Affiliations:** National Institute of Plant Genome Research, Aruna Asaf Ali Marg, New Delhi, 110067 India

## Abstract

Both phytohormones and non-coding microRNAs (miRNAs) play important role in root development in *Arabidopsis thaliana*. Mature miR166/165 s, which are derived from precursor transcripts of concerned genes, regulate developmental processes, including leaf and root patterning, by targeting *Class III HOMEODOMAIN LEUCINE-ZIPPER* (*HD-ZIP III*) transcription factors (TFs). However, their regulation through hormones remained poorly understood. Here, we show that several phytohormones dynamically regulate the spatio-temporal expression pattern of miR166/165 and target *HD-ZIP IIIs* in developing roots. Hormone signaling pathway mutants show differential expression pattern of miR166/165, providing further genetic evidence for multilayered regulation of these genes through phytohormones. We further show that a crosstalk of at least six different phytohormones regulate the miR166/165, their target *HD-ZIP IIIs*, and *KANADI* (*KANs*). Our results suggest that *HD-ZIP IIIs* mediated root development is modulated both transcriptionally through phytohormones and *KANs*, and post-transcriptionally by miR166/165 that in turn are also regulated by the phytohormonal crosstalk.

## Introduction

Phytohormones and several TFs play major role in root growth and development in *Arabidopsis*. Among these factors, several growth regulators known as phytohormones play important role during root growth and development^1, 2^. Phytohormones, such as auxin (Indole-3-acetic acid, IAA), gibberellic acid (GA), cytokinins (CKs), abscisic acid (ABA), jasmonic acid (JA), salicylic acid (SA), brassinosteroid (BR), etc. regulate various plant developmental processes by modulating the expression of downstream genes. Auxin is known as the master regulator of plant development, as it controls almost every developmental process in plants, including root development^3, 4^. Apart from auxin, GA also controls root growth by regulating cell elongation and proliferation through DELLA protein degradation^5^. CK acts antagonistically to auxin in root development by modulating the expression of auxin efflux carriers PIN-FORMED (PINs)^6^. ABA negatively regulates both primary and lateral root (LR) development^7^. Exogenous application of JA inhibits primary root growth at all concentrations, but induces LR formation at low concentrations whereas reduces at higher concentration^8^. SA also regulates root development in *Arabidopsis* ^9^. Root development involves gene regulation by a complex crosstalk among different hormone signaling pathways^10^.

Besides role in other developmental processes, several miRNAs have also been reported to regulate root development in *Arabidopsis* ^11^. An evidence of link between phytohormones and miRNAs was identified by the study of *hyponastic leaves1* (*hyl1*) mutant, which is defective in miRNA biogenesis and display abnormal response to different phytohormones such as ABA, auxin, and CK^12^.

Spatio-temporal expression pattern of genes largely contributes to their developmental role. Spatio-temporal expression or transcriptional pattern of different miRNA species of a family as well as their targets depends on the *cis*-regulatory elements, including hormone response elements, in their respective promoters. Therefore, to understand miRNA function it is necessary to decipher their upstream regulation. miR165 and miR166 are the two miRNAs that differ by only one nucleotide in their mature sequence and both target transcripts of same *HD-ZIP III* gene family members^13^. *Arabidopsis* genome encodes seven *MIR166* (*MIR166A*–*G*) and two *MIR165* (*MIR165A-B*), which finally produce same mature miR166 and miR165, respectively. miR166/165 regulates diverse developmental processes including shoot apical meristem (SAM) maintenance, leaf polarity, floral development, and root development in plants by negatively regulating *HD-ZIP III* gene family members - *PHABULOSA* (*PHB*), *PHAVOLUTA* (*PHV*), *REVOLUTA* (*REV*), *ARABIDOPSIS THALIANA HOMEOBOX 8* (*ATHB8*) and *ARABIDOPSIS THALIANA HOMEOBOX 15* (*ATHB15*)^14–17^. Recently, miR166/165 has been shown to be involved in root development in *Arabidopsis* ^18, 19^. *KANADI* genes (*KAN1 - KAN4)*, members of GARP transcription factors family show expression pattern complementary to *HD-ZIP IIIs* and also regulate *HD-ZIP IIIs* expression and function^20^. Genetic analyses revealed that *HD-ZIP IIIs* and *KAN* genes act antagonistically to each other to regulate organ patterning^21, 22^. Both miR166/165 and *KAN* genes are involved in maintaining abaxial fate of lateral organs by restricting the expression of *HD-ZIP IIIs* in the adaxial domain^21^. Although mature miR166 derived from all the seven *MIR166* genes are same, each individual *MIR166* gene exhibits specific spatio-temporal regulation^14^. Thus, the precise spatio-temporal expression of seven *MIR166* genes is possibly controlled by their transcriptional regulation in specific cell-types at specific stages of development.

Spatio-temporally regulated expression dynamics of a miRNA gene family is likely to regulate the expression pattern or accumulation of their target transcripts at post-transcriptional level. We have previously shown that promoter sequences of seven *MIR166* genes are significantly dissimilar to each other indicating the variations in the *cis-*regulatory elements in their promoters^23^. In the present study, we have analyzed the effects of various phytohormones on the expression pattern of miR166/165 s (precursors and mature), their target *HD-ZIP IIIs* and *KAN* genes during root development. We here show that various phytohormones influence the spatio-temporal expression pattern of all these genes, which often is due to their transcriptional regulation. We provide evidence that it is the dose of functional *HD-ZIP III* transcripts, regulated both at transcriptional and post-transcriptional level, which plays important role in root development. Our results uncover a complex hormonal crosstalk regulating *HD-ZIP IIIs* transcript level (at transcriptional/post-transcriptional level) by modulating the expression of miR166/165, *KANs* and hormone signaling during root development.

## Results

### Phytohormones dynamically regulate the expression pattern of miR166/165 in roots

Phytohormones such as auxin (IAA), GA, CK, JA, etc. regulate various aspects of plant growth and development^24^. Before analyzing hormonal regulation of miR166/165, we performed the growth analysis of wild type (Col-0) *Arabidopsis* roots in response to six phytohormones - IAA, GA, BAP, ABA, JA, and SA to test the reproducibility of phenotype in our growth conditions (Supplemental Fig. S1). Consistent with previous reports, our results confirmed that root growth and development are affected by aforesaid phytohormones.

**Figure 1:**
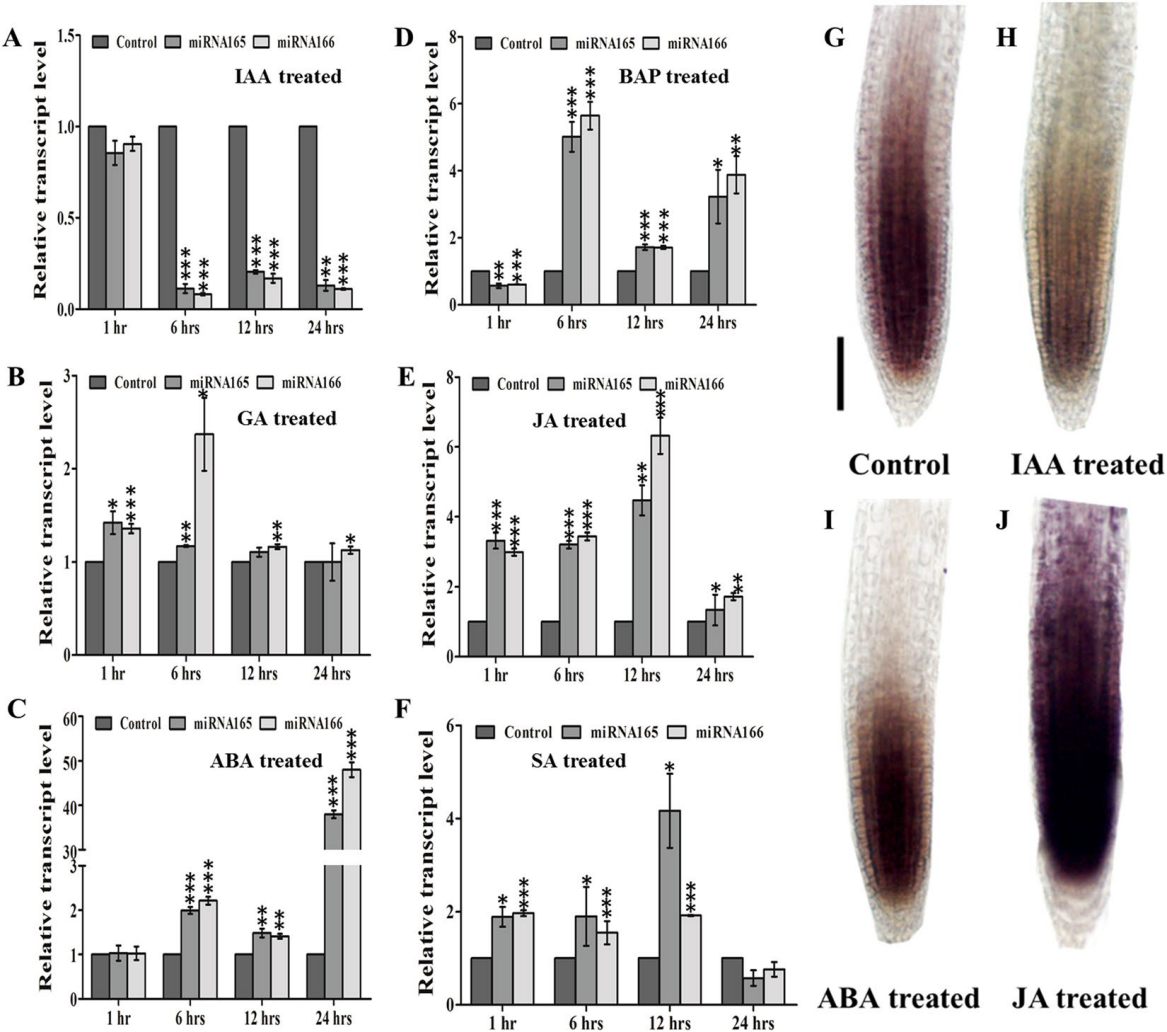
Phytohormones affect the expression level of mature miR166/165. The expression level of miR166/165 was quantified using stem-loop qRT-PCR in roots at 1, 6, 12, and 24 hrs after treatment with (**A**) 10 µM IAA, (**B**) 10 µM GA, (**C**) 10 µM ABA, (**D**) 10 µM BAP, (**E**) 20 µM JA, and (**F**) 100 µM SA. Whole mount *in situ* localization of miR166/165 was performed in 7 dag roots following 12 hrs of phytohormone treatment (**G–J**). (**G**) Control (without any treatment), (**H**) 10 µM IAA, (**I**) 10 µM ABA and with (**J**) 20 µM JA. *In situ* localization of miR166/165 in BAP, GA and SA treatment has been provided in Supplemental Fig. S3. Error bars indicate ± SE of two independent biological experiments. One-way ANOVA was performed. Statistically significant differences are indicated as, * for P < 0.05, ** for P < 0.01 and *** for P < 0.001. Scale bar indicates 50 µm.

To understand whether phytohormones regulate the expression of miR166/165 and contribute to miR166/165 mediated root development, we analyzed the expression of miR166/165 upon treatment with aforesaid phytohormones at different time intervals (1, 6, 12, and 24 hrs). Treatment with 10 µM IAA significantly reduced the level of mature miR166/165 at each time point, except at 1 hr of the study, as observed in stem loop qRT-PCR analysis (Fig. 1A). Whole mount *in situ* hybridization experiment illustrated the reduced accumulation of miR166/165 in IAA treated roots than control roots (Fig. 1G,H). GA treatment induced the expression level of miR166/165 at all time points, showing highest upregulation up to 2.5 fold at 6 hrs of treatment (Fig. 1B). After 12 and 24 hrs of GA treatment, the expression level of miR166/165 remained higher than the untreated control but lower than that of 6 hrs of the treatment (Fig. 1B). However, the whole mount *in situ* localization experiment illustrated the increased accumulation of miR166/165 after the treatment with GA at 12 hrs (Supplemental Fig. S3A,C). Treatment with 6-Benzylaminopurine (BAP or CK) induced the expression level of miR166/165 at each time points, except at 1 hr, where miR166/165 level was reduced in comparison to the wild type. After 1 hr, the expression of miR166/165 was induced in BAP treatment, whereas highest increase upto 5 fold was observed at 6 hrs of treatment (Fig. 1D). The whole mount *in situ* localization experiment showed the increased accumulation of miR166/165 after the treatment with BAP at 12 hrs (Supplemental Fig. S3A,B). ABA treatment led to no significant change in the expression of miR166/165 after 1 hr. After 6, 12, and 24 hrs of ABA treatment, the expression level of mature miR166/165 was upregulated by 2.2, 1.5 and 37–40 folds, respectively (Fig. 1C). Whole mount *in situ* localization experiment also confirmed the stem-loop qRT-PCR results exhibiting increased accumulation of miR166/165 upon ABA treatment (Fig. 1G,I). JA treated roots showed increased expression of mature miR166/165 at 1, 6, 12, and 24 hrs of the treatment (Fig. 1E). Upon SA treatment, the expression of mature miR166/165 was induced at 1, 6 and 12 hrs. However, the expression of miR166/165 was downregulated after 24 hrs of the treatment (Fig. 1F). The whole mount *in situ* localization showed increased accumulation of miR166/165 in 7 dag roots treated with JA for 12 hrs in comparison to control (Fig. 1G,J). Localization of miR166/165 in SA treated 7 dag roots at 12 hrs showed slight difference in expression pattern to that of qRT-PCR (Supplemental Fig. S3A,D). We cannot rule out that this difference might be due to fluctuation and stability of mature miR166/165 among treatment points and/or the sensitivity of two techniques. The tissue used during qRT-PCR includes whole root (both primary root and LR) which may lead to the change in expression pattern between qRT-PCR and whole mount *in situ*. These results suggest that phytohormones dynamically regulate the expression of miR166/165 during stages of root development.

### Differential expression of *MIR166* precursors contributes to the altered level of miR166/165 level in response to hormones

**Figure 2:**
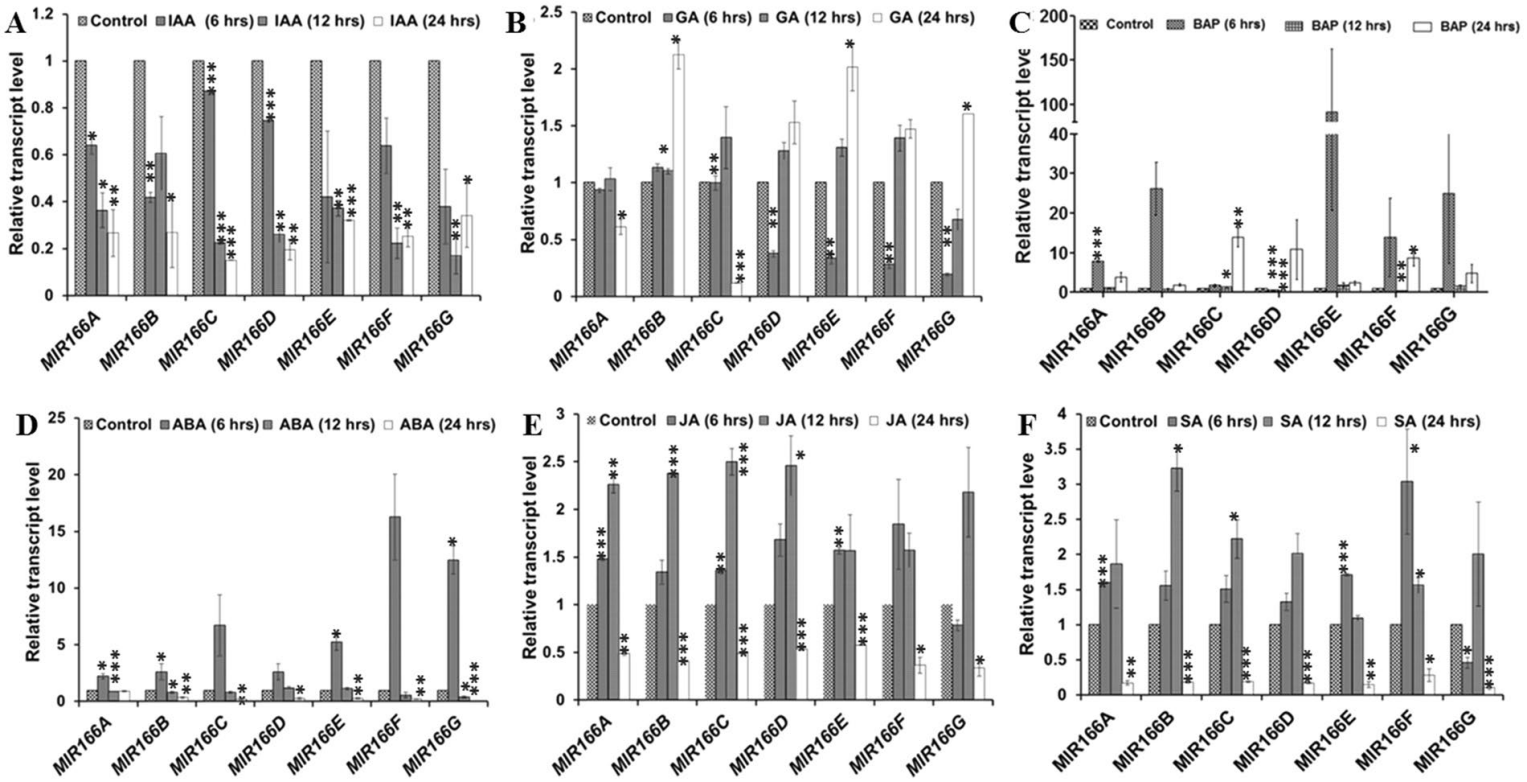
Phytohormones affect the expression level of precursor genes of *MIR166*. The expression level of seven *MIR166(A*–*G)* genes was quantified using qRT-PCR in roots at 1, 6, 12, and 24 hrs with hormone concentrations of (**A**) 10 µM IAA, (**B**) 10 µM GA, (**C**) 10 µM BAP, (**D**) 10 µM ABA, (**E**) 20 µM JA and (**F**) 100 µM SA. The control indicates without treatment in each case. Error bars indicate ± SE of two independent biological experiments. One-way ANOVA was performed. Statistically significant differences are indicated as, * for P < 0.05,** for P < 0.01 and *** for P < 0.001.

We observed that phytohormones affect the expression of mature miR166/165 and root development. We analyzed the effect of hormone treatment on transcriptional regulation of *MIR166A–G* genes at different time points (6, 12 and 24 hrs) by qRT-PCR (Fig. 2). Interestingly, exogenous application of 10 µM IAA led to the downregulation of *MIR166A*–*G* genes at all three time points (Fig. 2A). IAA treatment produced similar effect on the expression pattern of both mature miR166 and its precursors. Exogenous application of 10 µM GA induced the expression of *MIR166B* after 6 hrs of the treatment (Fig. 2B). We observed significant induction in the transcript levels of all *MIR166* genes, except *MIR166G*, after 12 hrs of treatment (Fig. 2B). The expression of all *MIR166* precursors except *MIR166A* and *MIR166C* was further induced by up to 1.5 folds and above at 24 hrs of GA treatment (Fig. 2B). Exogenous application of 10 µM BAP induced the expression of all *MIR166* members, except *MIR166D* after 6 hrs of treatment. After 12 hrs of BAP treatment, *MIR166A, C, E* and *F* were upregulated whereas *MIR166B, D* and *G* were downregulated (Fig. 2C). The expression level of *MIR166A*–*G* was induced at 24 hrs of treatment (Fig. 2C). Treatment with 10 µM ABA induced the expression of all precursors by 2 to 15 folds as compared to the control, immediately after 6 hrs of the treatment (Fig. 2D). At 12 hrs of the ABA treatment, *MIR166D* and *MIR166G* showed increased expression, *MIR166A* expression remained unchanged and *MIR166B, C, F, G* expression levels were reduced in comparison to untreated control (Fig. 2D). At 24 hrs of ABA treatment, the expression of *MIR166B-G* decreased to almost undetectable level, except for *MIR166A*. The application of 20 µM JA and 100 µM SA induced the expression of *MIR166A-F* except *MIR166G* at 6 hrs of the treatment, which was detected below that of untreated control (Fig. 2E,F). The transcript level of all precursors further increased as compared to the untreated control at 12 hrs of JA and SA treatment (Fig. 2E,F). Finally, the expression of all the precursors was reduced below the untreated control at 24 hrs of JA and SA treatment (Fig. 2E,F). These differential expression patterns of seven *MIR166* precursors at different time intervals of treatment indicated their temporal transcriptional regulation by developmentally important phytohormones.

### Mutants defective in hormone signaling show altered expression of miR166/165

**Figure 3:**
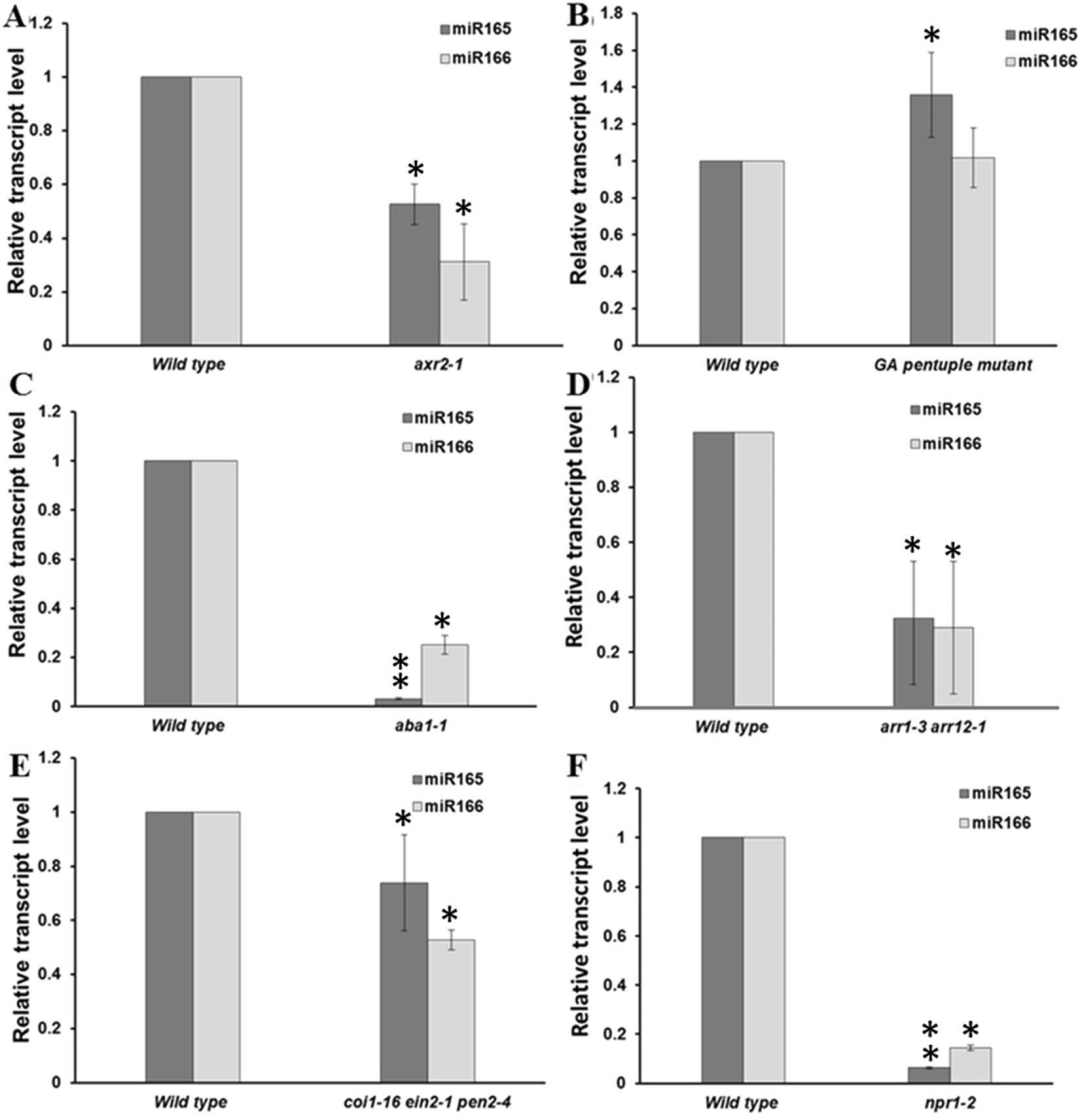
The expression of miR166/165 was affected in hormone insensitive mutants. The expression level of miR166/165 was quantified using stem-loop qRT-PCR in (**A**) *axr2-1* mutant. (**B**) GA insensitive pentuple (*gai-t6 rga-t2 rgl1-1 rgl2-1 rgl3-1*) mutant. (**C**) *aba1-1* mutant. (**D**) *arr1-3 arr12-1* mutant. (**E**) *coi1-16 ein2-1 pen2-4* mutant. (**F**) *npr1-2* mutant. Error bars indicate ± SE of two independent biological experiments. One-way ANOVA was performed. Statistically significant differences are indicated as, * for P < 0.05,** for P < 0.01 and *** for P < 0.001.

To confirm the transcriptional regulation of miR166/165 through phytohormones, we analyzed the expression of miR166/165 in hormone insensitive mutants. Stem-loop qRT-PCR results showed that the exogenous IAA treatment led to the downregulation of miR166/165 expression level. To further confirm our result, we checked the expression of miR166/165 in seedlings of IAA insensitive *auxin resistant2-1* (*axr2-1*) mutant, which is defective in auxin perception and root development^25^. Interestingly, we observed that *axr2-1* mutant root had significantly reduced level of miR166/165 expression (Fig. 3A). The reduced expression of miR166/165 in *axr2-1* could be the result of increased endogenous auxin in *axr2-1*, as observed in case of *shy2-2* ^26^.

Stem-loop qRT-PCR results showed that the exogenous GA treatment led to the upregulation of miR166/165 level. To further confirm our result, we checked the expression of miR166/165 in roots of GA insensitive pentuple mutant of all five DELLA proteins, *gai-t6 rga-t2 rgl1-1rgl2-1rgl3-1* using stem-loop qRT-PCR analysis. When we checked the expression of miR166/165 in DELLA pentuple mutant, we found increased expression level of miR166/165, similar to the exogenous GA treatment (Fig. 3B).

The exogenous ABA application inhibited primary root growth (Supplemental Fig. S1C). We observed significant induction of miR166/165 expression by ABA treatment in comparison to wild type. To confirm genetically, we further checked the expression of miR166/165 in *aba1-1* mutant, an ABA biosynthetic mutant, which is ABA deficient^27^. Roots of *aba1-1* mutant showed reduced expression level of miR166/165 in comparison to wild type (Fig. 3C).

To confirm cytokinin mediated regulation of miR166/165, we checked the expression of miR166/165 in *arr1-3 arr12-1* double mutant, which shows CK insensitivity towards root elongation, LR formation and callus induction^28^. Since exogenous CK inhibits primary root elongation, *arr1 arr12* mutant shows longer root phenotype suggesting CK insensitivity^29^. In our experiment, exogenous BAP treatment induced the expression of miR166/165. In comparison to wild type, the expression of miR166/165 was reduced in *arr1 arr12* mutant, which further confirmed the CK mediated regulation of miR166/165 (Fig. 3D).

The exogenous JA treatment caused reduction in primary root length (Supplemental Fig. S1D). We observed that JA treatment induced the expression of miR166/165 significantly. To genetically confirm this, we checked the expression of miR166/165 in seedlings of *coi1-16 ein2-1 pen2-4* triple mutant, which is JA insensitive^30^. Triple mutant roots showed reduced expression of miR166/165 in comparison to wild type (Fig. 3E).

Exogenous SA application showed inhibitory effect on primary root length (Fig. 3F). Exogenous application of SA induced the transcript level of miR166/165 in roots. To genetically confirm this, we checked its expression in *npr1-2* mutant, which is insensitive to SA treatment. The endogenous SA level in *npr1-2* mutant was less in comparison to wild type root^31^. Roots of *npr1-2* show reduced expression level of miR166/165 in comparison to wild type, which further confirmed SA mediated upregulation of miR166/165 in *Arabidopsis* roots.

### Over expression of miR166 (*miR166-Oe*) alters the sensitivity of roots to hormones

**Figure 4:**
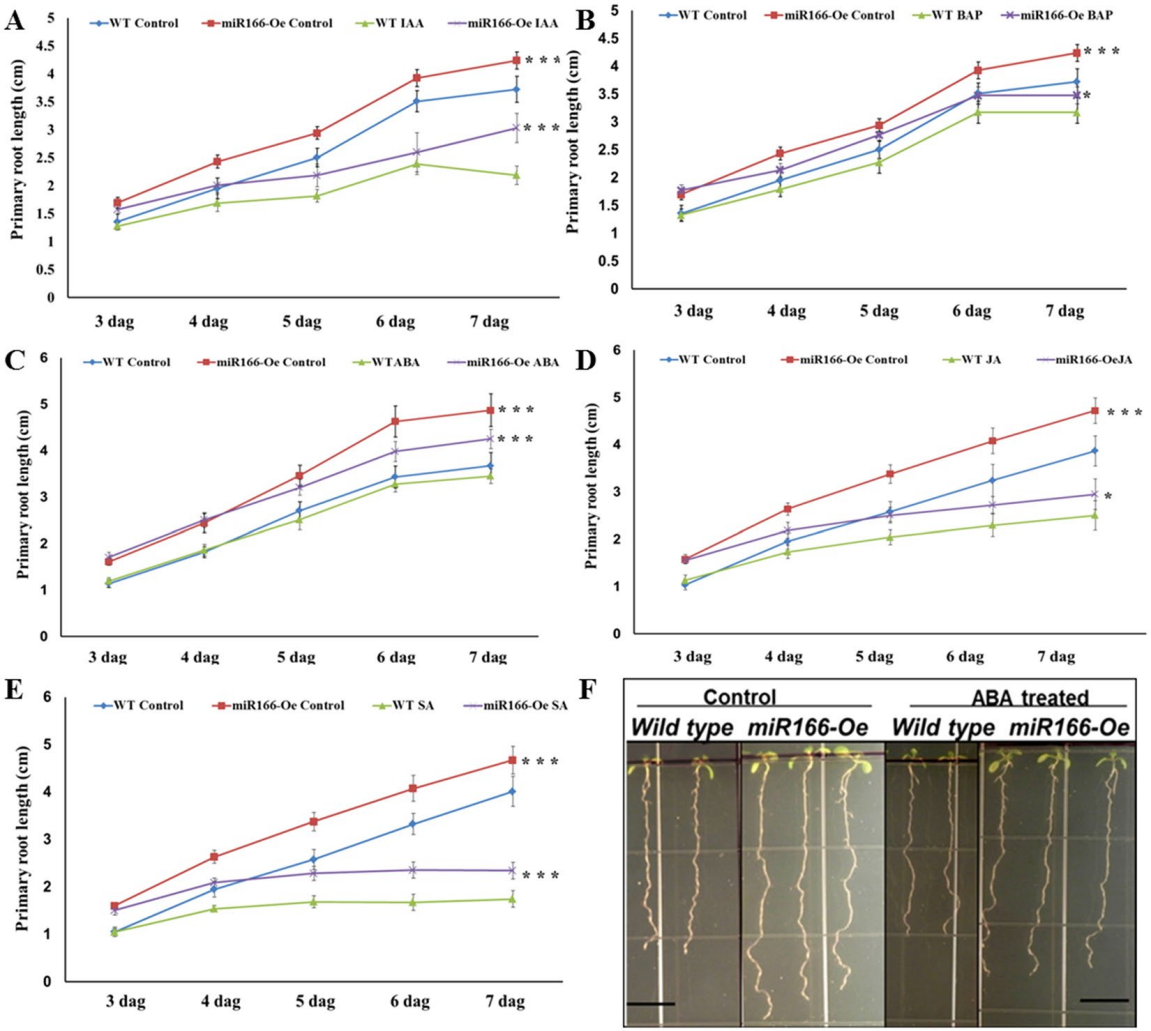
Phytohormones affect the root growth of *miR166-Oe* and wild type plants. Primary root growth of *miR166-Oe* in the presence of (**A**) 0.1 µM IAA, (**B**) 0.1 µM BAP, (**C**) 1 µM ABA, (**D**) 20 µM JA, and (**E**) 100 µM SA. The primary root length measurement was done using Image J software from 3 dag to 7 dag. Error bars indicate ± SD (n = 10). One-way ANOVA was performed. Statistically significant differences are indicated as, * for P < 0.05,** for P < 0.01 and *** for P < 0.001.

We have recently reported that *miR166-Oe* affects primary root growth through down regulation of *HD-ZIP IIIs* transcripts^19^. To investigate if the accumulation of miR166/165 and reduction in the expression of targets affected the response of roots to hormone treatment, we performed phenotypic analysis of wild type and *miR166-Oe* plants treated with different phytohormones (Fig. 4). The treatment with 0.1 μM IAA showed reduction in primary root length of both wild type and *miR166-Oe* plants in comparison to their respective untreated control, which suggest that *miR166-Oe* roots were less sensitive to IAA than wild type treated plants (Fig. 4A). We observed that primary root growth of *miR166-Oe* was more sensitive towards 1 μM ABA treatment than that of wild type plants (Fig. 4C). In order to investigate the effect of CK on root growth of *miR166-Oe* plants, we treated *miR166-Oe* line with 0.1 µM BAP and observed the phenotype. We found that primary root length of CK treated *miR166*-*Oe* plants was reduced as compared to wild type roots, suggesting that *miR166-Oe* plants are more sensitive to CK treatment (Fig. 4B). Additionally, primary root growth of *miR166-Oe* plants was more sensitive to 20 μM JA treatment (Fig. 4D). Upon treatment with 100 μM SA, less reduction in primary root length suggested that *miR166-Oe* plants are less sensitive to SA (Fig. 4E). Collectively these results suggest that the sensitivity of *miR166-Oe* plant roots vary in response to different phytohormone treatments, which could be the effect of downregulation of *HD-ZIP III*s by increased miR166/165 level in *miR166-Oe* plants.

### Phytohormones differentially regulate the expression of *HD-ZIP IIIs* during root development

**Figure 5:**
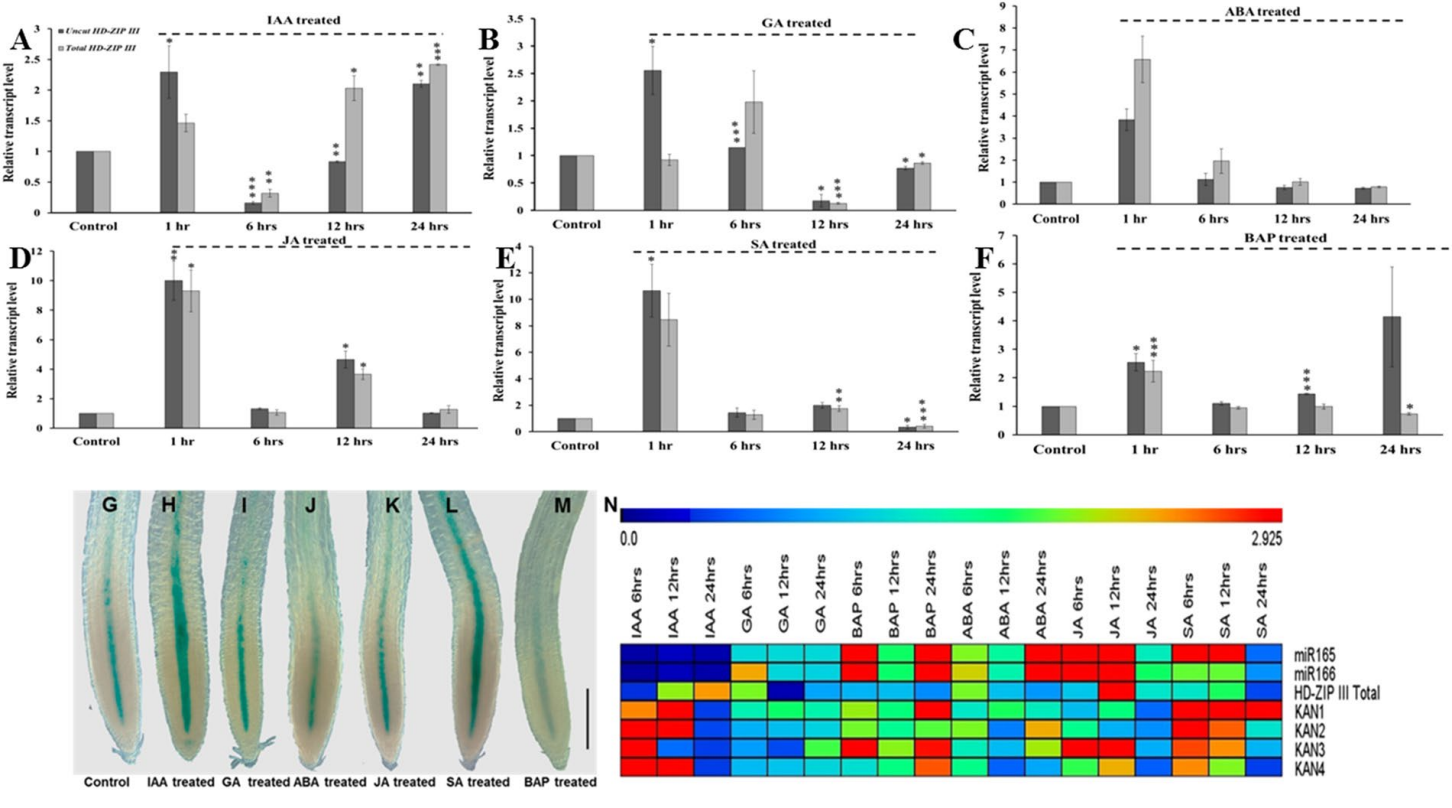
Phytohormones affect the expression level of *HD-ZIP IIIs* transcript level. The expression of total *HD-ZIP IIIs* at 1, 6, 12 and 24 hrs of the treatment of (**A**) 10 µM IAA, (**B**) 10 µM GA, (**C**) 10 µM ABA, (**D**) 20 µM JA, (**E**) 100 µM SA and (**F**) 10 µM BAP. The control indicates untreated roots in each case. Error bars indicate ± SE of two independent biological experiments. One-way ANOVA was performed. Statistically significant differences are indicated as, * for P < 0.05,** for P < 0.01 and *** for P < 0.001. (**G–M**) GUS expression analysis of pPHB::PHB-GUS roots after 12 hrs of the hormone treatment in (**G**) untreated control (**H**) 10 µM IAA, (**I**) 10 µM GA, (**J**) 10 µM ABA, (**K**) 20 µM JA, (**L**) 100 µM SA, and (**M**) 10 µM BAP.

Since miR166/165 cleaves and negatively regulates *HD-ZIP III* transcripts, differential root growth observed in *miR166-Oe* plants upon phytohormone treatment might be due to the altered expression levels of *HD-ZIP IIIs* ^19^. At cellular level, the transcript level of total *HD-ZIP IIIs* is ultimately the result of both transcriptional regulation and post-transcriptional cleavage by miR166/165. Therefore, we analyzed the accumulation of *HD–ZIP IIIs* transcript level in response to various phytohormone treatments at different time intervals (1, 6, 12 and 24 hrs) by real time qRT-PCR (Fig. 5). When we checked the early response of IAA on the expression of *HD–ZIP IIIs*, we found an induction of expression. At 6 hrs of the IAA treatment, the expression of total *HD-ZIP IIIs* was only 10% of the untreated control (Fig. 5A). IAA induced the expression of total *HD-ZIP IIIs* at 12 and 24 hrs after treatments, in comparison to untreated control (Fig. 5A). GA treatment induces the transcript of *HD-ZIP IIIs* at 1 and 6 hrs, however, it reduced total *HD-ZIP IIIs* transcript level at 12 and 24 hrs, in comparison to untreated control (Fig. 5B). The expression of uncleaved *HD-ZIP III*s was more at 1, 12 and 24 hrs, whereas total *HD-ZIP IIIs* transcript level was downregulated at all the time points, except at 1 hr. The expression of *HD-ZIP IIIs* upon ABA treatment was induced at 1 and 6 hrs, whereas at 12 and 24 hrs the transcripts of *HD-ZIP IIIs* were reduced (Fig. 5C). JA treatment induces the transcript of *HD-ZIP III* at 1 and 12 hrs, however, transcript levels were reduced at 6 and 24 hrs of treatment (Fig. 5D).

SA treatment induced the transcription of *HD-ZIP IIIs* at 1, 6 and 12 hrs, however, it reduced the expression of *HD-ZIP IIIs* at 24 hrs of treatment, in comparison to untreated control (Fig. 5E). Our results suggest that phytohormones regulate *HD-ZIP IIIs* at transcriptional level, besides post-transcriptional regulation through miR166/165 (Fig. 5N).

### Expression analysis of pPHB::PHB-GUS under treatment with different phytohormones confirms hormonal regulation of *HD-ZIP IIIs*

Since expression level of *HD-ZIP III* genes was affected by different phytohormones, subsequently affecting the root development, we studied the spatial expression pattern of *PHB* (a member *HD-ZIP IIIs*) reporter pPHB::PHB-GUS in response to phytohormones. The differential expression of pPHB::PHB-GUS (a translation fusion line) in hormone treated root represents the outcome of *in vivo* regulation of *HD-ZIP IIIs* through phytohormones. This suggests that the regulation of *HD-ZIP IIIs* through phytohormones was stable at protein level. We analyzed GUS expression in the root meristem after 12 hrs of hormone treatment (Fig. 5G-M). It was found that GUS expression was stronger and extended upwards in the primary root tip in IAA and SA treatments, as compared to the untreated controls (Fig. 5G,H,L). GUS expression analysis confirmed the qRT-PCR results illustrating the increased expression of overall total *HD-ZIP IIIs* at 12 hrs upon treatment with IAA and SA. We observed reduced GUS expression after 12 hrs of BAP treatment (Fig. 5M). BAP treatment of pPHB::PHB-GUS line confirmed our qRT-PCR results, as there was a reduction in the overall total *HD-ZIP IIIs* expression after 12 hrs of BAP treatment (Fig. 5M). We did not observe any significant difference in GUS expression pattern upon GA, ABA and JA treatment, although differential expression pattern of total *HD-ZIP IIIs* transcripts was found at 12 hrs of treatment with hormones (Fig. 5I,J,K). This could be the result of differential amplification of other *HD-ZIP IIIs* transcripts (other than *PHB*) in the qRT-PCR analysis. The significant increase in pPHB::PHB-GUS expression upon SA treatment further indicates potential role of SA signaling in *HD-ZIP IIIs* mediated root development, in a manner opposite to IAA.

### *KANs* show differential expression pattern in response to phytohormones

We showed that phytohormones regulate the expression of miR166/165 and its target *HD-ZIP IIIs*. *KAN* genes, members of GARP transcription factor family, regulate shoot development and organ patterning by antagonistically regulating the expression of *HD-ZIP IIIs*. Like leaves, the expression domain of both *HD-ZIP IIIs* and *KANs* are complementary to each other in roots^20^. Therefore, we also checked the effect of phytohormones on *KAN* genes during root growth.

**Figure 6:**
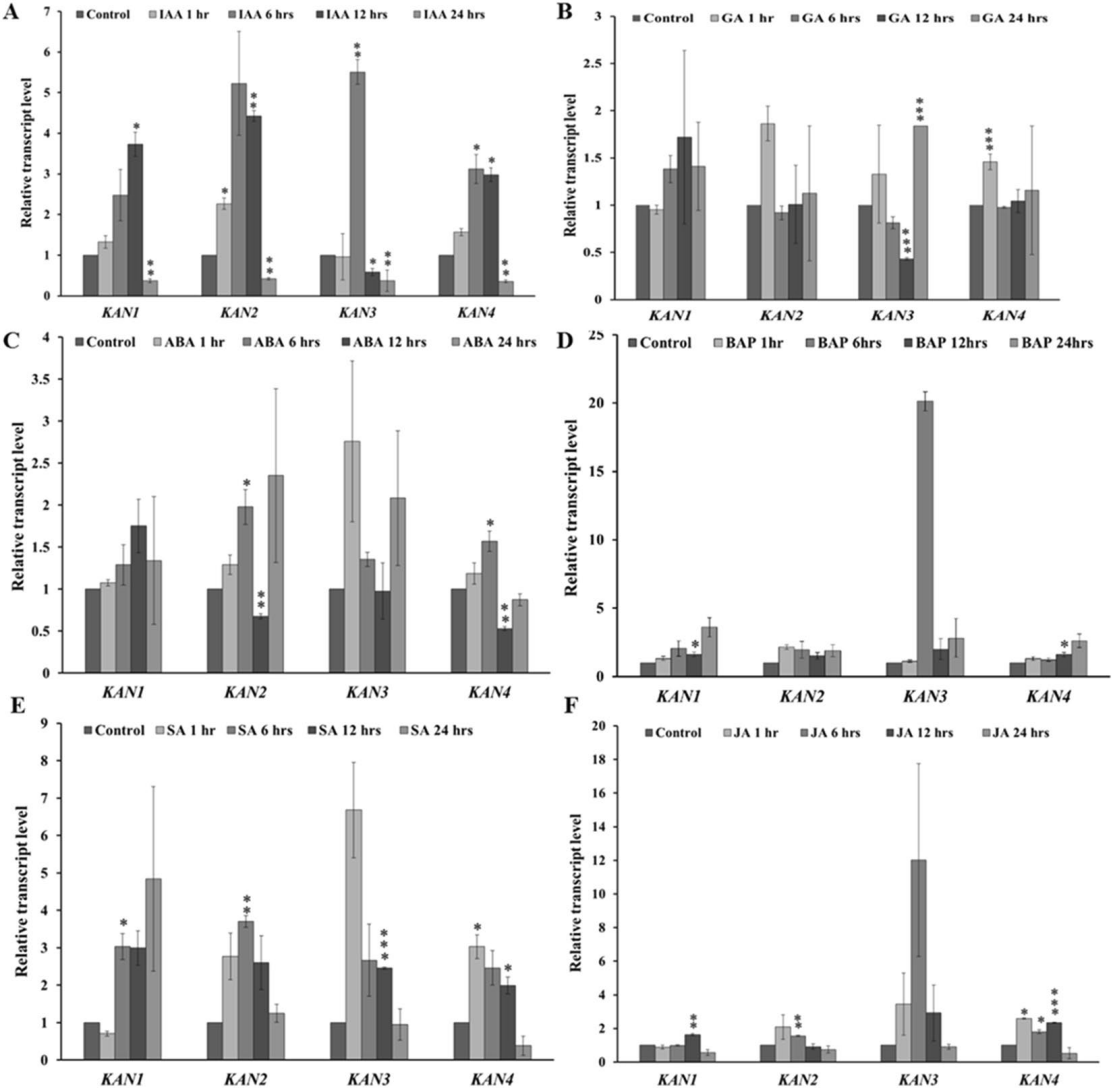
Phytohormones affect the expression level of *KAN* genes. The expression of *KAN* genes in *Arabidopsis* roots was checked at 1, 6, 12 and 24 hrs of the treatment with (**A**) 10 µM IAA, (**B**) 10 µM GA, (**C**) 10 µM ABA, (**D**) 20 µM JA, (**E**) and 100 µM SA. The control indicates without hormone treatment in each case. Error bars indicate ± SE of two independent biological experiments. One-way ANOVA was performed. Statistically significant differences are indicated as, * for P < 0.05,** for P < 0.01 and *** for P < 0.001.

We analyzed the accumulation of transcripts of *KAN* gene family members, *KAN1*, *KAN2*, *KAN3*, and *KAN4*, in response to hormone treatments for 1, 6, 12 and 24 hrs (Fig. 6). During IAA treatment, the expression of *KAN1*, *KAN2*, and *KAN4* was found to be upregulated at 1, 6 and 12 hrs of the treatment up to 2.5 to 5 folds (Fig. 6A). At 24 hrs, the expression of all the four *KANs* was significantly downregulated (Fig. 6A). The expression of *KAN* genes did not change significantly in response to GA treatment at any time point. Among all the *KAN* genes, the expression of only *KAN1* remained higher than the untreated control at all the time points, except at 1 hr (Fig. 6B). The expression of the *KAN* gene family members showed differential expression in response to ABA treatment with immediate increase at 1 and 6 hrs, followed by decrease at 12 hrs, and again increase at 24 hrs (Fig. 6C). The dynamic change in the expression of *KAN* genes may be due to the presence of an ABA mediated secondary regulatory mechanism. BAP treatment reduced overall expression level of total *HD-ZIP IIIs* at each time point, suggesting a linear temporal regulation of transcription. On the other hand, BAP treatment induced the expression level of both *KANs* and miR166/165 at each time point, suggesting their linear temporal regulation by CK signaling in root at transcriptional and post-transcriptional level (Figs 5F and 6D). The expression of all *KAN* genes was induced at 1, 6 and 12 hrs of SA treatment (Fig. 6E). However, treatment with SA for 24 hrs induced the expression of only *KAN1* (Fig. 6E). JA treatment for 1 and 6 hrs induced the expression of all *KANs*, except *KAN1* (Fig. 6F). JA treatment for 12 hrs induced the expression of all *KANs*, except *KAN2*. At 24 hrs of the treatment, the expression of all *KAN* genes was downregulated in comparison to wild type. Differential expression pattern of *KAN* genes suggests transcriptional regulation of *KANs* through phytohormones, which in turn would regulate transcription of *HD-ZIP IIIs* (*Fig.* *5N*).

## Discussion

### Phytohormone signaling regulates expression of miR166/165

To study the hormone dependent regulation of miR166/165 expression, we analyzed the expression level of miR166/165 in response to different phytohormones. We found significant decrease in the expression and accumulation of mature miR166/165 as well as their precursors upon IAA treatment (Figs 1A and 2A). Auxin is a major regulatory hormone in plants and several reports have established a complex miRNA mediated crosstalk between auxin and other phytohormone signaling^32^. This indicates that the reduction in the expression level of miR166 in response to auxin, as evidenced by qRT-PCR and *in situ* localization results, is possibly affected by a complex crosstalk with other hormones (Fig. S3). Expression of miR166/165 was induced and expression of target *HD-ZIP IIIs* was downregulated after treatment with BAP, ABA and JA, which suggests their transcriptional regulation in root through phytohormone signaling (Figs 1 and 5). Mature miR166/165 s are derived from the precursors of their respective *MIR166/165* genes, which showed differential expression pattern in response to phytohormone treatment indicating their transcriptional regulation through phytohormones.

### Genetic evidences using hormone signaling mutants confirm hormonal regulation of miR166/165

Various hormone insensitive mutants showed differential expression pattern of miR166/165, which further confirmed their hormonal regulation (Fig. 3). Hormone insensitive mutants used in the present study are deficient in respective phytohormone endogenously, hence the expression patterns of miR166/165 was opposite to that of exogenous hormone treatment. In case of GA, CK, ABA, JA, and SA treatment, expression and accumulation of mature miR166/165 was found to be upregulated at 12 hrs of the treatment (Fig. 1B–F). Opposite expression of miR166/165 in hormone insensitive mutants, as shown by our qRT-PCR and whole mount *in-situ* hybridization experiments, further suggests their transcriptional regulation through phytohormones (Fig. 1 and Supplemental Fig. S3).

### Overexpression of miR166/165 mediated reduction of *HD-ZIP IIIs* alters the sensitivity of roots towards phytohormones

We have previously described that primary root elongation in *miR166*-*Oe* is promoted by down regulation of *HD-ZIP IIIs* ^19^. Therefore, we analyzed the root growth of *miR166*-*Oe* in response to various phytohormones (Fig. 4). The response of *miR166-Oe* roots to various phytohormones indicates a correlation between levels of miR166/165 and *HD-ZIP IIIs* transcripts and their transcriptional regulation through phytohormones. Treatment with IAA and CK resulted in reduced root length in *miR166-Oe* plants, suggesting the possible involvement of miR166/165 target *HD-ZIP IIIs* in IAA and CK dependent root growth. In previous reports, it has been shown that *HD-ZIP IIIs* genes act downstream of auxin, as indicated by upregulated *ATHB8* expression in shoot vasculature after auxin treatment^33^. Tryptophan dependent auxin biosynthesis is required for *HD-ZIP IIIs* expression^16^. This finding suggests that the effect of IAA on root growth in *miR166-Oe* plants is the result of altered expression of *HD-ZIP IIIs* genes through both transcriptional and post-transcriptional regulation. The activity of *PHB* and *PHV* is required for *ISOPENTENYLTRANSFERASE 7* (*IPT7*) expression and CK biosynthesis and thus regulate root growth^34^. CK in turn represses the expression of *PHB* in a feedback mechanism, which was also supported by our pPHB::PHB-GUS expression result (Fig. 5M)^34^. Thus, the effect of CK on root growth of *miR166-Oe* plants could be due to the reduced activity of *PHB* and *PHV*. Treatment of *miR166-Oe* plants with other phytohormones like ABA, JA and SA also leads to change in root growth (Fig. 4). Expression of mature miR166/165 was upregulated upon ABA treatment and *miR166-Oe* roots were more sensitive to ABA treatment, suggesting possible involvement of miR166/165 in ABA mediated stress responses through negative regulation of *HD-ZIP IIIs* transcript level. JA and SA are known to be involved in pathogen responses in plants^35^. Our results have shown that the expression of miR166/165 is upregulated upon JA and SA treatment, suggesting that miR166/165 may play role in biotic and abiotic stress responses through the regulation of *HD-ZIP IIIs* transcript level. Our results also suggest that root growth is affected by transcriptional regulation of *MIR166s* and *HD-ZIP IIIs* genes modulated by hormones, besides post-transcriptional regulation of *HD-ZIP IIIs* by miR166/165.

### *HD-ZIP IIIs* transcript level depend on post-transcriptional regulation by miR166/165 and transcriptional regulation by *KANs* and phytohormones

miR166/165 cleaves *HD-ZIP III* transcripts and differential root growth observed in *miR166-Oe* plants upon phytohormone treatment might be the result of altered expression levels of *HD-ZIP IIIs* ^19^. In addition to miR166/165, *KAN* genes also regulate the expression of *HD-ZIP III* genes in an antagonistic manner^22^. It is also likely that *HD-ZIP IIIs* and *KANs* themselves are transcriptionally regulated by multiple hormones and result in a feedback regulatory circuit with miR166/165.

We showed that the expression of total *HD-ZIP IIIs* remained relatively unaltered at 1 hr of auxin treatment, however, auxin treatment for 6 hrs repressed the expression. Although auxin did not induce miR166/165 expression at 6 hrs of the treatment, the expression of *KANs* was induced by 2.5 to 5 folds, which could downregulate *HD-ZIP IIIs* transcription (Fig. 5A). This indicates that an indirect regulation by *KANs* may contribute to the reduction in the expression of *HD-ZIP IIIs* genes in response to auxin treatment. The total transcripts of *HD-ZIP IIIs* genes increased by up to 2 folds at 12 hrs of IAA treatment (Fig. 5A), which correlates with the reduced expression of miR166/165 s (Fig. 1A). A further increase of the total transcript levels of *HD-ZIP IIIs* by up to 2.5 folds at 24 hrs may be largely due to the reduced expression and activity of miR166/165 and *KAN* genes (Fig. 5A). The expression of total *HD-ZIP IIIs* remained unaltered immediately after 1 hr of GA treatment, which, however, induced the expression by 2 fold at 6 hrs of treatment (Fig. 5B). Although the expression of miR166/165increased up to 2.3 fold at 6 hrs of the treatment, the upregulation of *HD-ZIP IIIs* genes could largely be due to their transcriptional induction upon GA treatment. The level of total *HD-ZIP IIIs* transcripts decreased at both 12 and 24 hrs of the GA treatment, in comparison to the untreated control (Fig. 5B). The expression of *KAN* genes remained relatively unaltered by GA treatment at all the three time points, suggesting no direct transcriptional regulation of *KANs* by GA. Additionally, ABA treatment induced the expression of *HD-ZIP IIIs* genes at 1 and 6 hrs of the treatments (Fig. 5C). However, ABA reduced the expression of *HD-ZIP IIIs* nearly equivalent to the control at 12 and 24 hrs of treatment, which could be caused by significant increase in the transcript level of miR166/165 at the same time points (Fig. 1C). ABA treatment also induced the expression of *KANs* showing their regulation by ABA (Fig. 6C). BAP treatment reduced the expression level of total *HD-ZIP IIIs* at each time points, except at 1 hr, suggesting differential temporal regulation of *HD-ZIP IIIs* through CK. BAP treatment induced the expression level of both *KANs* and miR166/165 at all the time points, suggesting their linear temporal regulation by CK signaling in root (Figs 5F and 6D). In case of JA treatment, the transcript level of total *HD-ZIP IIIs* was 4 fold upregulated at 1 and 12 hrs whereas, at 6 and 24 hrs the transcript level of total *HD-ZIP IIIs* was similar to that of wild type. The nearly similar level of total *HD-ZIP IIIs* transcripts in comparison to the control at 6 hrs of JA treatment correlates with the increased level of both miR166/165 and *KANs* (Figs 5D and 6F). However, sudden increase in the overall transcripts of *HD-ZIP IIIs* genes up to 4 fold at 12 hrs could be the result of their own transcriptional induction via JA (Fig. 5D). At 24 hrs of treatment, insignificant changes in total *HD-ZIP IIIs* transcript correlate to increased level of miR166/165 and *KANs* (Figs 5D and 6F). Treatment with SA showed induction of both *HD-ZIP IIIs* genes and miR166/165 after 1 and 6 hrs of the treatment followed by a further increase at 12 hrs (Fig. 5E). This could be due to their independent and strong transcriptional activation upon SA treatment. At 24 hrs of the treatment, nearly unchanged level of the total *HD-ZIP III*s transcripts correlates with the level of miR166/165, which might be below the control and insufficient to reduce *HD-ZIP III* transcripts post-transcriptionally (Fig. 5E). Thus, it seems that SA regulates the transcription of both the gene family to the similar extent. The transcript level of total *HD-ZIP IIIs* was induced at 6 and 12 hrs of SA treatment, however, at 24 hrs of treatment, the transcript level was significantly reduced in comparison to wild type (Fig. 5E). SA also showed strong transcriptional regulation for *KANs*, as evident by the induced expression level at 6 and 12 hrs of SA treatment, however, the expression of only *KAN1* was found to be upregulated at 24 hrs of treatment (Fig. 6E).

In a recent report it was shown that *KAN1* inhibits auxin biosynthesis, transport and signaling in a manner opposite to that of *HD-ZIP IIIs* ^36^. Gain-of-function mutant *phb-1d* produced shorter primary roots than wild type plants and it was shown that *kan1 kan2 kan3* triple mutant displayed phenotype similar to *phb-1d* ^36^. Auxin was shown to upregulate the expression of *HD-ZIP IIIs* (Fig. 5G,H)^37^. Therefore, we hypothesize that KANs regulate root growth by auxin mediated regulation of *HD-ZIP IIIs* expression. We showed that the treatment with auxin led to the upregulation of *HD-ZIP IIIs* and *KAN* expression thus disturbing the KAN mediated regulation of auxin signaling, which lead to shorter roots (Supplemental Fig. S1A). Together, our results suggest that auxin dependent balanced activity of both *KAN* and *HD-ZIP IIIs* are required for proper root development. This strengthens our hypothesis that miR166/165 mediated post-transcriptional regulation of the transcript level of *HD-ZIP IIIs*, altered expression of *KANs* and a crosstalk between hormones cumulatively lead to altered root growth in response to phytohormones.

We observed a significant increase in the GUS expression of pPHB::PHB-GUS line upon SA treatment. We also showed that the level of total *HD-ZIP IIIs* transcripts was induced upon SA treatment. Although IAA and SA act antagonistically to each other^38^, we found increased expression of pPHB::PHB-GUS in both IAA and SA treatment (Fig. 5H,L). We showed that both SA and IAA induced the expression of miR166/165 (Fig. 1F). Thus, SA treatment induced the expression of both miR166/165 and its target *HD-ZIP IIIs* genes. Among six phytohormones analyzed in our study, IAA, CK, JA, and SA are the strongest potential transcriptional regulators of *MIR166s*. In contrast to other phytohormones, IAA negatively regulates expression of miR166/165.

We showed that CK treatment induced the expression level of both miR166/165 and *KANs*, whereas reduced the expression of total *HD-ZIP IIIs*. Decrease in the expression level of total *HD-ZIP IIIs* and induced expression of miR166/165 upon BAP treatment indicate a complex transcriptional and post-transcriptional regulation of *HD-ZIP IIIs* by CK. Both KANs and HD-ZIP IIIs work antagonistically by modulating the function of genes involved in auxin signaling, biosynthesis and transport, whereas the CK signaling component works in feedback loop with *HD-ZIP IIIs* ^34^. Therefore, the variation observed in the expression pattern of *KANs* and *HD-ZIP IIIs* at each time points after BAP treatment suggest their complex transcriptional regulation through a crosstalk of phytohormones signaling. JA negatively regulates root growth, which is also evident by studies with *coi1-1*, a JA mutant showing pleiotropic developmental defects and inhibition of root growth^39^.

**Figure 7:**
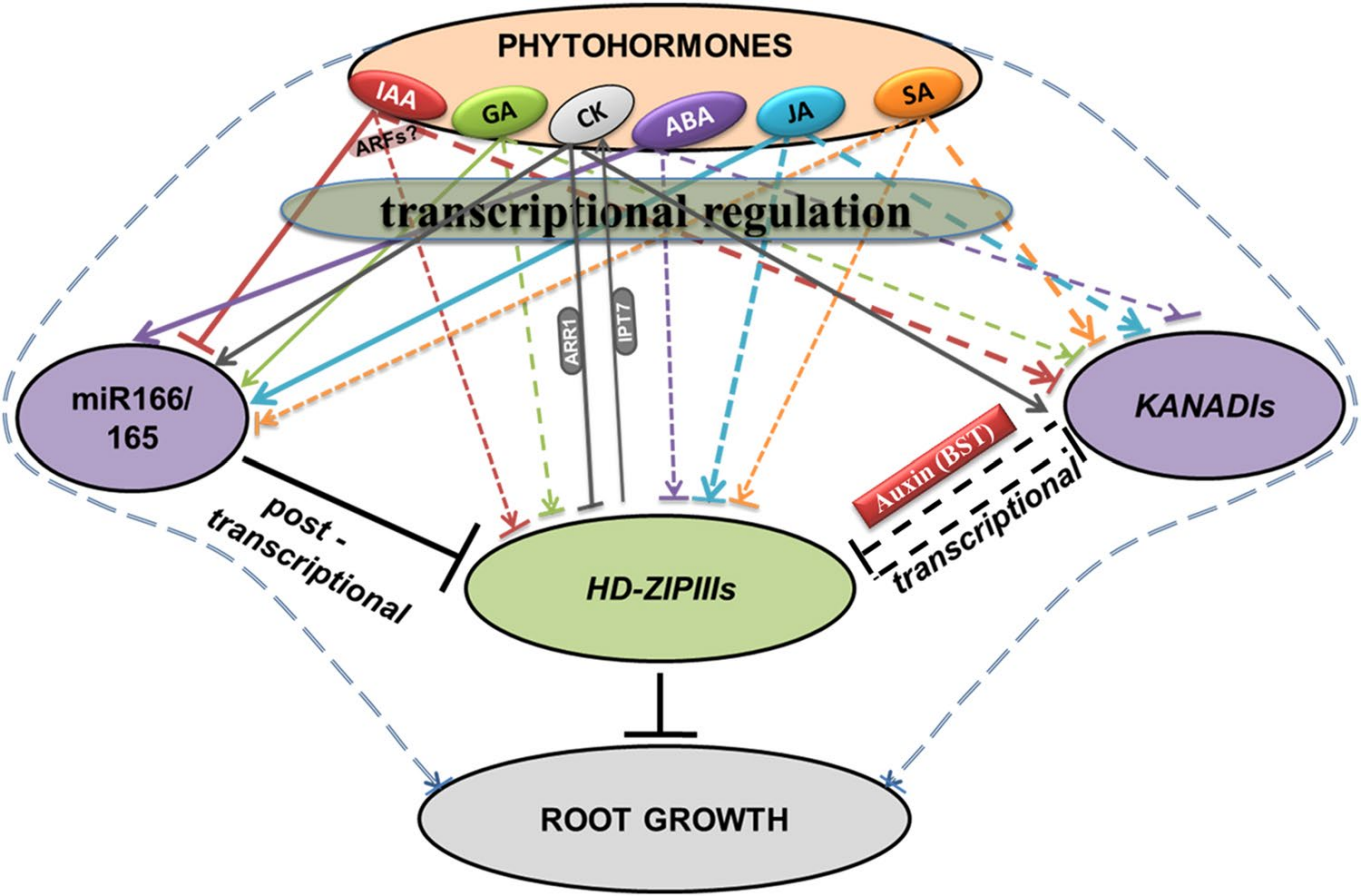
A hypothetical model showing complex crosstalk among phytohormones, miR166/165, *HD-ZIP IIIs* and *KAN* genes regulating root growth. miR166/165 and *HD-ZIP IIIs* mediated root growth and development is regulated by various hormones such as IAA (red lines), GA (green lines), CK (grey lines), ABA (purple lines), JA (blue lines) and SA (orange lines) through transcriptional regulation of miR166/165, *HD-ZIP IIIs* and *KAN* genes. CK mediated regulation was adapted from^34^. miR166/165 post transcriptionally regulates *HD-ZIP IIIs* expression, whereas *KANs* and *HD-ZIP IIIs* regulate each other transcriptionally through auxin biogenesis, signaling, transport components^36, 37^. Solid thick lined arrows indicate strong transcriptional induction of genes. Solid thick lines with stop bars indicate strong transcriptional repression of genes. Narrower lined arrows/stop bars represent moderate or weak induction/repression, respectively. Dotted lines with arrow and repression mark represent both induction and repression of expression. Two dotted double lines (blue) with arrow/stop bars indicate hormonal regulation of root growth independent of miR166/165 and *HD-ZIP IIIs*.

Based on these results, we proposed a model showing complex hormonal regulation of miR166, *HD-ZIP IIIs* and *KANs*, which advocates that these genes act simultaneously during root development, involve some feedback regulation, and thus regulate root growth in *Arabidopsis* in a complex regulatory network (Fig. 7). Taken together, our results, in line with previous reports, suggest that miR166/165 post-transcriptionally regulate *HD-ZIP IIIs*, which are also regulated by various phytohormones and *KANs* at transcriptional level. A hormonal crosstalk along with feedback regulation by *KANs* and *HD-ZIP IIIs*, and miR166/165 maintain the balance of functional transcript level of *HD-ZIP IIIs* and phytohormone signaling module, which are critical for maintaining proper root growth. Increasing bodies of evidences suggests huge functional conservation and some level of diversification of miRNAs and their respective targets across plant species^40^. Therefore, it would be interesting to study the conservation and diversification of phytohormone mediated regulation of miR166/165, its target *HD-ZIP IIIs* across the plant species. Since both nutrient availability and phytohormones are known to regulate both miRNA expression and root development, it raises the possibility of regulation of these miRNAs through nutrient-phytohormone crosstalk, which remains an interesting area to be addressed^1, 41^.

## Methods

### Plant materials, growth and treatment conditions

*Arabidopsis thaliana* (ecotype Col-0 or L*er*) and transgenic plants in either of these backgrounds were used for various experiments. Overexpression of miR166/165 (*miR166-Oe*) has been described earlier^19^. Most seed materials used in experiments were obtained from *Arabidopsis* Biological Resource Centre (ABRC). Growth assay was performed as described previously^42^. To analyze the effect of hormones on the root growth, 7 days old wild type plants were transferred onto the half strength Murashige and Skoog (MS) agar medium supplemented with phytohormones at different time point 1 hr, 6 hrs, 12 hrs and 24 hrs^5, 43, 44^. Mutants used in the study were *axr2-1* (CS3077), *gai-t6 rga-t2 rgl1-1rgl2-1rgl3-1* (CS16298), *aba1-1* (CS21), *arr1-3 arr12-1* (CS6981), *coi1-16 ein2-1 pen2-4* (CS67818), *npr1-2* (CS3801), which were first confirmed for homozygosity using PCR-based genotyping and analyzed for phenotype.

### RNA extraction and qRT-PCR analysis

Total RNA was isolated from the root tissues using TRI-reagent at different time points (Sigma-Aldrich, St. Louis, MO, USA)^45^. RNA isolation and qRT-PCR for the transcript level of *MIR166* gene family, *HD-ZIP IIIs* and *KAN* gene family were done as described previously^19, 46^. Primers specific for each of the seven *MIR166* (*A*–*G*) precursors were designed as described earlier^47^. For the analysis of transcript level of uncleaved or non-targeted *HD-ZIP IIIs*, primers were designed in coding regions flanking miR166/165 target sites. For the analysis of overall or total transcripts of all *HD-ZIP IIIs* genes, primers were designed from the conserved sites of the 5’ region of the cleavage site. The detail of primers is given in Table S1. The transcript level was normalized using *ACTIN4 (ACT4)* as endogenous control.

### Validation of the mature miR166/165 expression profile via stem-loop qRT-PCR

The expression profile of mature miRNAs was analyzed by stem-loop qRT-PCR as described previously^48^. Sequence of the primers used in the reaction is given in Table S1.

### Whole mount *in situ* hybridization

Whole mount *in situ* hybridization was performed with modification in the method described previously^49–51^. In brief, 7 days old seedlings were transferred to control and hormone plates and after 12 hrs, the treated seedlings were used for *in situ* hybridization using locked nucleic acid (LNA) probe complementary to miR166/165 (Eurogentec, Belgium). Plant tissues/samples were fixed in 1:1 mixture of paraformaldehyde based fixative (in PBS, phosphate buffer saline) and n-Heptane for 45 minutes, followed by washing twice with 100% methanol and ethanol. Tissues/samples were rehydrated by passing through gradients of ethanol (100%, 70%, 50%, and 25%) and for 10 minutes each and followed by treatments with fixative (20 minutes) and (PBS; 2 × 10 minutes). Root samples were kept for pre-hybridization for 1 hr in a hybridization mixture (50% formamide, 5X SSC, 0.1 mg/ml heparin, and 0.1% tween-20), followed by hybridization with DIG (digoxigenin)-labelled LNA probe overnight. LNA probe was labeled using DIG-Oligonucleotide 3’end leveling kit, 2^nd^ generation (Roche Diagnostics, India), as per company’s manual. After hybridization, root samples were washed in wash buffers (containing 50% formamide, 2X SSC and 0.1% Tween-20; same without formamide, and with reduced SSC), and were incubated in anti-DIG antibody (Roche Diagnostics, India; dilution of 1:2000 times) for 4 - 12 hrs, after washing with PBT (PBS plus 0.1% Tween 20). After antibody treatment, samples were again washed with PBT, followed by incubation in detection buffer (100 mM, Tris-HCl, pH 9.5, 50 mM MgCl_2_, 100 mM NaCl). Detection was done by incubating with NBT/BCIP based detection solution (Roche Diagnostics, India), as per company’s manual. After visible signal was observed, the reaction was stopped with 10% glycerol, roots were mounted on slides with 10% glycerol and imaged using Nikon 80i microscope. Sequence of the miR166 LNA probe is provided in Supplemental Table S1.

## Electronic supplementary material

Supplementary information
