# Supplementary material for: Phytohormonal crosstalk modulates the expression of miR166/165s, target Class III HD-ZIPs, and KANADI genes during root growth in Arabidopsis thaliana
Source: Sci Rep. 2017 Jun 13;7:3408. doi: 10.1038/s41598-017-03632-w (PMC5469759; doi:10.1038/s41598-017-03632-w)
Supplement: Supplementary file 1 — Supplementary information [file 41598_2017_3632_MOESM1_ESM.pdf]

Title of the article:

Phytohormonal crosstalk modulates the expression of miR166/165s, target *Class III HD-ZIPs,* and *KANADI* genes during root growth in *Arabidopsis thaliana*

Full names of authors:

Archita Singh[1$], Shradha Roy[1$], Sharmila Singh[1$], Shabari Sarkar Das[1], Vibhav Gautam[1], Sandeep Yadav[1], Ashutosh Kumar[1], Alka Singh[1], Sukanya Samantha[1] and Ananda K. Sarkar[1,*]

Supplemental Information: Figure 3, Table 1

Supplementary Figure:

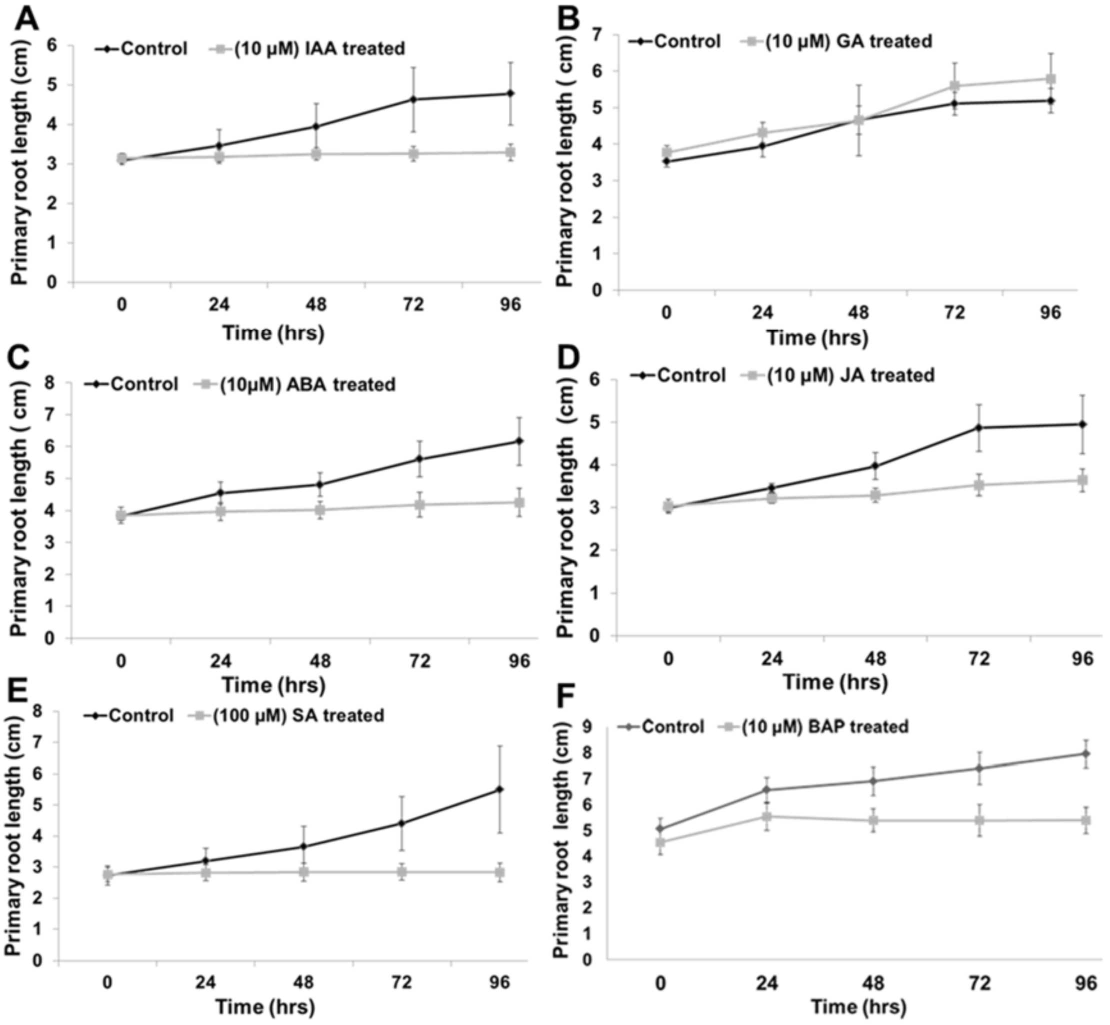

Fig. S1. Phytohormones affect the root growth of *Arabidopsis thaliana*.

The primary root length of wild type plants at 7 dag was measured from 0 hr to 96 hrs after hormone treatment at five time points. (A) 10 µM IAA, (B) 10 µM GA, (C) 10 µM ABA, (D) 20 µM JA, (E) 100 µM, and (F) 10 µM BAP. Primary root length measurement was done using Image J software. Error bars indicate ± SD (n = 10). One-way ANOVA was performed. Statistically significant differences are indicated as *, for $P < 0.05$,** for $P < 0.01$ and *** for $p < 0.001$.

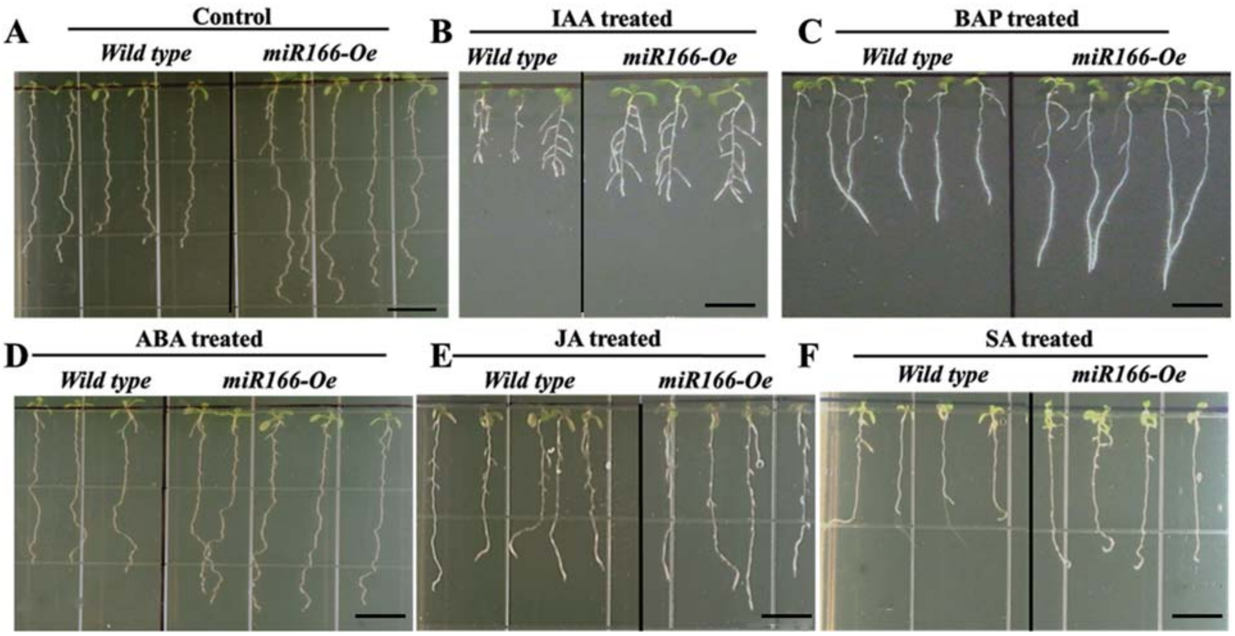

Fig. S2. Phytohormones affect the root growth pattern of wild type and *miR166-Oe*. Root growth phenotype of *miR166-Oe* and wild type plants at 7 dag. (**A**) Control, (**B**) 0.1 μM IAA, (**C**) 0.1μM BAP, (**D**) 1 μM ABA, (**E**) 20 μM JA, and (**F**) 100 μM SA. Black line indicates scale bar of 2 cm.

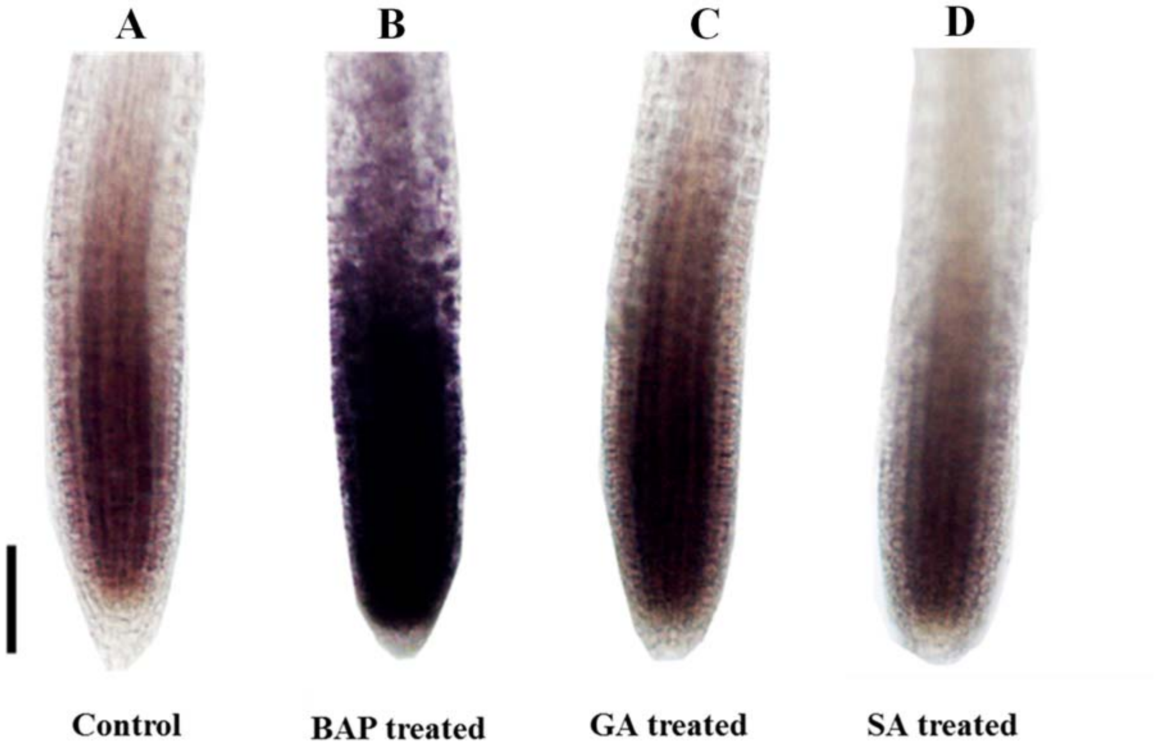

Fig. S3. Whole mount *in situ* localization of miR166/165 in roots (7 dag) treated with phytohormones for 12 hrs. The expression pattern (purple/brown staining) of miR166/165 in (**A**) Control root without hormone treatment, which is same as in Fig.1, as part of the same experiment, (**B**) 10 μM BAP, (**C**) 10 μM GA, and (**D**) 100 μM SA. Scale bar indicates 50 μm.

Supplemental Table S1. List of primers used in the study.

S.No.	Primer Name	Primer Sequence	Purpose
1	*MIR166a*FP	GACTCTGGCTCGCTCTATTCA	qRT-PCR
2	*MIR166a*RP	TGGTCCGAAGACGCTAAAAC	qRT-PCR
3	*MIR166b* FP	ATCATTCTCTTCATCATCACCA	qRT-PCR
4	*MIR166b* RP	CCCTCTTTAAATCCTCTTCTTCT	qRT-PCR
5	*MIR166c* FP	ACATACCTTTCTTTCTCTTCTTCT	qRT-PCR
6	*MIR166c* RP	CAAGACTAGAACCACGTTATCA	qRT-PCR
7	*MIR166d* FP	GAGGAATATTGTCTGGCTC	qRT-PCR
8	*MIR166d* RP	TGGTCCGAGAATCATTTAG	qRT-PCR
9	*MIR166e* FP	GGAATGTTGTCTGGCACGA	qRT-PCR
10	*MIR166e* RP	GGAATGAAGCCTGGTCCGA	qRT-PCR
11	*MIR166f* FP	GGTGAATGATGCCTGGCTC	qRT-PCR
12	*MIR166f* RP	GGAATGAAGCCTGGTCCGA	qRT-PCR
13	*MIR166g* FP	TAGGGTTTAGAGGAATGTTGTT	qRT-PCR
14	*MIR166g* RP	CGATAAAATAGGTTGAGGGGA	qRT-PCR
15	*KAN1* FP	GATCCAGCATTCAAAATCAGG	qRT-PCR
16	*KAN1* RP	TCCAAATTGATCAAGAAAGTCA	qRT-PCR
17	*KAN2* FP	TTTGCATGGGAAGTTAATCG	qRT-PCR
18	*KAN2* RP	TTGTTCCCGAGATGCTTGAT	qRT-PCR
19	*KAN3* FP	GTTTTAATCTCCACAACCG	qRT-PCR
20	*KAN3* RP	GCTCAAGTACTGATTTTGGG	qRT-PCR
21	*KAN4* FP	GGTAGAGAAAGAGGCAGAGCAGAG	qRT-PCR
22	*KAN4* RP	TTGTCCTTAGTGTGATGAGTTGTTCC	qRT-PCR
23	miR165F1	CGGCGGTCGGACCAGGCTTCA	Stem-loop qRT-PCR
24	miR165SL1	GTTGGCTCTGGTGCAGGGTCCGAGGTATTCGCACCA GAGCCAACGGGGG	Stem-loop qRT-PCR
25	miR166F1	CGGCGGTCGGACCAGGCTTCA	Stem-loop qRT-PCR
26	miR166SL1	GTTGGCTCTGGTGCAGGGTCCGAGGTATTCGCACCA GAGCCAACGGGGA	Stem-loop qRT-PCR
27	UvRP1	GTGCAGGGTCCGAGGT	Stem-loop qRT-PCR
28	*HD ZIP IIIs* (Uncut) FP	GAGTTCATTTCCAAGGCGAC	qRT-PCR
29	*HD ZIP IIIs*(Uncut) RP	CAAGGCTTATCCTTTAGGATCTC	qRT-PCR
30	*HD ZIP IIIs*(Total) FP	GACTGTCCTAAACCGAGCTC	qRT-PCR
31	*HD ZIP*	AGCGGTCATTCTCTTCCATC	qRT-PCR

	IIIs(Total) RP		
